# A multidisciplinary study unveils the nature of a Roman ink of the I century AD

**DOI:** 10.1038/s41598-021-86288-x

**Published:** 2021-03-31

**Authors:** Mirta Sibilia, Chiaramaria Stani, Lara Gigli, Simone Pollastri, Alessandro Migliori, Francesco D’Amico, Chiara Schmid, Sabina Licen, Matteo Crosera, Gianpiero Adami, Pierluigi Barbieri, Jasper R. Plaisier, Giuliana Aquilanti, Lisa Vaccari, Stefano Buson, Federica Gonzato

**Affiliations:** 1 Nuclear Science and Instrumentation Laboratory, Physics Section, International Atomic Energy Agency (IAEA), Seibersdorf, Austria; 2 CERIC-ERIC, Strada Statale 14, 34149 Basovizza, Trieste, Italy; 3 Elettra-Sincrotrone Trieste S.C.P.A, Strada Statale 14, 34149 Basovizza, Trieste, Italy; 4 Department of Engineering and Architecture, University of Trieste, Via A. Valerio, n 6/a, 34127 Trieste, Italy; 5 Department of Chemical and Pharmaceutical Sciences, University of Trieste, Via L. Giorgieri, 1, 34127 Trieste, Italy; 6 Museo Nazionale Atestino, via G. Negri 9/c, 35042 Este, PD Italy

**Keywords:** Chemistry, Materials science, Physics

## Abstract

A multi-instrumental approach combining highly sensitive Synchrotron Radiation-based techniques was used to provide information on the real composition of a dry black ink powder found in a bronze inkwell of the first century AD. The presence of Pb, Cu and Fe in the powder, revealed by XRF and ICP-OES data, leads to raise several hypotheses on their origin. The inkpot and its lid were also investigated by Hand-Held XRF, revealing a bronze alloy (Cu-Sn) with a certain amount of Fe and Pb. The lid was found to be particularly enriched in lead. XRPD, XAS and FTIR measurements showed a substantial presence of silicates and common clay minerals in the ink along with cerussite and malachite, Pb and Cu bearing-carbonates, respectively. These evidences support the hypothesis of an important contamination of the ink sample by the burial environment (soil) and the presence of degradation products of the bronze inkpot. The combined use of IR, Raman, and GC-MS evidenced that the black ink is mainly composed of amorphous carbon deriving from the combustion of organic material mixed with a natural binding agent, Arabic gum.

## Introduction

Writing was essential for the development of civilization. It allowed people to keep records, transmit and store information relatively easily and to spread them across a large area and also across the time. Archaeology shows that writing began with inscriptions scratched into the surface of stone, clay or wax tablets, and bark (3500–3000 BCE). At a later stage, with the introduction of papyrus, parchment and, later, paper, writing became an issue of depositing contrast materials on the writing support using brushes, reeds, pens and similar means. These contrast materials are generically called inks. The term comes from the Greek “*enckauston*”*,* meaning burnt, cooked. In fact, it is well documented that throughout antiquity and, at least until the IV century AD^1^, black inks were mainly based on amorphous carbon obtained from soot (“lamp black”), charcoal, or bone black^2,3^. The amorphous carbon was usually dissolved in a binder mixed with a small amount of water. The most common binder used was Arabic gum from Acacia *nilotica*, but the use of other kinds of binders, such as glair or animal glue, cannot be ruled out^2^. These carbon-based inks, probably the most ancient inks known, were used by humans since 2500 BC and cited in medieval recipes up to the thirteenth century AD^1^.

During the IV century AD, a new kind of ink, called iron-gall ink, emerged. It was obtained by mixing gall-nuts, iron or copper metal sulphates, water and Arabic gum. From the early Middle Ages onwards, it became the most common ink in the history of the western world^1^. However, some recent studies on the chemical composition of inks, already spread on their ancient writing supports (papyri, parchment or paper)^4–8^, have changed this perspective. Brun et al. identified the so-called “mixed ink” on some ancient Herculaneum papyri by Synchrotron Radiation (SR) X-Ray Fluorescence (XRF). This ink was produced by intentionally mixing soot or charcoal with metallic compounds, such as lead-based minerals, possibly added to enhance the ink black pigmentation^6,7^.

Moreover, employing a multi-analytical approach, Christiansen et al.^2^ found that the black and red inks used on Egyptian papyri from the Tebtunis temple library also belong to “mixed inks”, containing Cu and both Fe and Pb, respectively. The same author, performing a detailed investigation by means of highly sensitive SR µ-XRF and µ X-ray Absorption Near Edge Structure (µ-XANES), discovered that the copper-containing black carbon ink on some Egyptian papyri, coming from different time periods and geographical regions, was related to the use of soot obtained as by-products of metallurgical processes^2^. This shows that a multi-analytical approach is required for analyses of ancient artifacts of Cultural Heritage due to their complex nature. As a matter of fact, only a multi-technique approach allows distinguishing the original materials from the environmental contaminations and from the degradation products due to the weathering processes of the burial environment. Furthermore, a correct interpretation of the results requires a close cooperation among archaeometrists, physicists, geologists, chemists and archaeologists, museum curators and conservators. SR facilities represent the place where this synergetic work can take place due to the presence of researchers with different backgrounds along with the possibility to exploit SR advantages to get high-quality experimental data. This means reduced dwell times, reduced spot sizes, and higher signal-to-noise ratio of the measurements, allowing the investigation of unique and precious artifacts in a non–invasive and non-destructive way.

In this work we studied the chemical composition of a dry, black powder found in a bronze inkwell of the first century AD. According to a stylistic study of the inkwell and its attested use during the Roman period^9,10^, we assumed the powder to be an ink. The importance of the present work mainly resides in the rare possibility to directly analyze the inkwell content, avoiding the interference from any kind of writing support. Unfortunately, the inkwell lid was found to be lifted around 45 degrees from the container. Although an accurate restoration and microstratigraphic excavation allowed the archaeologists and conservators to distinguish between soil and ink remains, contaminations cannot be excluded a priori. In order to unravel the original composition of our ink powder, an exhaustive investigation was performed. The analysis was conducted exploiting several analytical methods, both non-destructive and destructive, based and not on SR, working at different spatial scales, for elemental, molecular and structural analysis, as detailed in the Methods section. Each technique was selected with the aim to answer the questions progressively raised during the investigation of this complex and possibly contaminated sample. Through the integration of complementary results, we were able to obtain a complete overview of the ink, determining its original recipe and distinguishing it from all the possible contamination sources and degradation products. In fact, we verified that, not only the interference of the burial environment, but also the prolonged contact of the black powder with the bronze inkwell, contributed to the alteration of the original formula of the ink. For this reason, also the container was investigated to evaluate the origin of eventual metallic contaminations in the black powder. It also allowed gaining more insight on the metallurgic technique of bronze manufacturing during the Roman period and its degradation products.

The combination of such a huge number of analytical techniques allowed us to avoid any kind of misinterpretation of the results.

## The archaeological context

**Figure 1:**
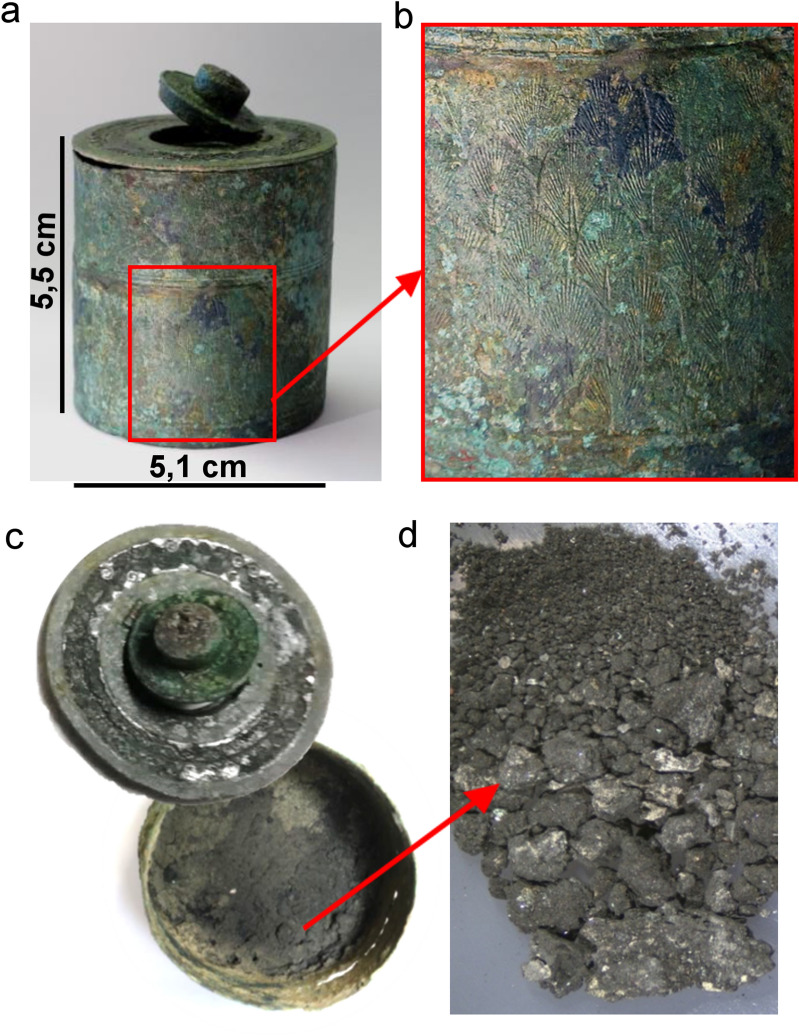
(**a**) the inkpot; (**b**) the inkwell bronze decoration; (**c**) an internal view of the inkwell with the black powder on the bottom; and a top view of the lid: traces of silver agemina are clearly visible; (**d**) the black powder with colored particles.

The inkpot IG 15,352, shown in Fig. 1, is part of the grave goods of the civic tomb XII—Palazzina Capodaglio—1878. It belongs to the collection of the Museo Nazionale Atestino of Este, Padua, Italy and was discovered during the excavation of a cemetery in the locality of Morlungo, Palazzina-Capodaglio, in the municipality of Este by Francesco Soranzo in 1878^11^. (For the complete grave good, see Supplementary Fig. S1 online).

The cemetery was established on the paleochannel of an ancient river and the tomb was found at a depth of 1.20 m. It was a simple shaft tomb without any lithic structure surrounding. The bronze inkwell, recently restored, well represents the Type Noll (Fig. 1a). It is made of a copper-tin alloy decorated with inlaid silver (“agemina”) on the lid (Fig. 1c). It can be associated with the n. 43 discovered in Trieste, Italy (decorated in gold niello)^9^. The body is decorated with several rows of overlapping palmettes (Fig. 1b), and the lid with a two facing bands of a running scroll motif. The lid has a circular aperture in the middle, closed by a small double cylindrical disk attached to the lid by a hinge. The closing mechanism consists of a small spring contained inside the upper cylinder which allowed to move a small piston anchored in a hole placed in the collar of the lower cylinder. The bottom is stamped with the name *“CARTILIUS”*. The same stamp appears on good quality fibulae produced in northern Italy^12^.

The inkwell is very similar to the one recovered in Nida^9^. It represents one of the rare examples found in Italy with these characteristics and is complete in all its elements. The presence of inkwells in grave goods is well documented in all of Europe but it is quite rare in Italy and Spain, in particular for type Noll. As reported by Eckardt^13^, the presence of the inkwells in graves acted as a symbol of skills, social practice and important forms of cultural knowledge, suggesting that the deceased belonged to a very high social class.

## Results and discussion

From here on, we will discuss in detail the analyses performed on the inkwell and its content, the specific role of each applied method and the complementarity of the obtained results that allowed determining the final ink powder composition as summarized in Table 1.

**Table 1:** Summary of the ink composition, as revealed by the different methods applied for the analysis, and attribution of the specific constituents to original ink composition, corrosion products and soil contamination.

Phase	Formula	Method	Attribution
Amorphous carbon	C	RAMAN, GC–MS	INK
Arabic gum	Polysaccharides + Glycoproteins	IR, GC–MS	INK
Cerussite	PbCO3	XAS, XRPD, IR	Corrosion product/soil contamination
Malachite	Cu_2_(OH)_2_CO_3_	XRPD, IR, SEM	Corrosion product/soil contamination
Fe and Cr Oxides (possibly as Chromite)	Fe2^+^Cr2O4	SEM	Corrosion product
Anglesite	PbSO4	XAS	Degradation product/soil contamination
Quartz	SiO2	XRPD, IR	Soil contamination
Albite	NaAlSi_3_O_8_	XRPD, IR	Soil contamination
Illite	(K,H_3_O)(Al,Mg,Fe)_2_ (Si,Al)_4_O_10_	XRPD, IR	Soil contamination
Oligoclase	(Na,Ca)(Si,Al)_4_O_8_	IR	Soil contamination

### Inkwell characterization

The alloy composition of the inkwell was investigated by means of a Hand-Held X-Ray Fluorescence (HHXRF) spectrometer. Several points of both the inkwell body (externally, on the side wall and on the bottom) and of the lid were analyzed. The measurements revealed that the container is mainly constituted by a Cu-Sn alloy with an average value of 69.4 and 20.7% respectively and by a certain amount of Pb and Fe, with an average value of 2 and 3.9%, respectively (Table 2). These values match well with those reported by literature ^14^. It is indeed well-documented that, during the Roman period a small amount of Pb could be added to the bronze alloy. In the manufacturing of sheets and wires, to avoid cracking during forging and hammering, not more than 1–2% of Pb should be added. Higher percentages of Pb could be added to produce heavier, larger or more complex castings. In such cases, the material is referred to as leaded bronze alloy^14^. It has to be also highlighted that HHXRF pointed out a non-homogeneous Pb distribution. Indeed, with respect to the main body, the lid is much enriched in Pb, showing an average amount of 13.4%. Some authors^15,16^ report that several inkwells have been discovered to have a copper-alloy body and leaded lid, since a heavy lid could preserve the ink solution from fast drying. Other elements, such as Ti, P, V, Cr, Mn, Co, Ni, Zn, Zr and Mo, have been detected in trace amounts in the alloy of the container. As reported by Martinòn-Torres et al.^17^, they can be considered as impurities of the metallurgic process of the ancient bronze. The “niello” decoration on the lid was also analyzed confirming the presence of silver.

**Table 2:** Percentages of the main metals revealed both in the inkwell and ink powder by HHXRF, ICP-OES, SR-XRF, SR-XRPD.

		Cu (%)	Sn (%)	Pb (%)	Fe (%)	Ca (%)	As (%)
HHXRF	Lid	49.6 (2)	28.6 (2)	13.4 (1)	2.7 (1)	–	–
	Inkwell	69.4 (3)	20.7 (1)	2.0 (1)	3.9 (1)	–	–
ICP–OES	Ink	15.0*	ND	3.5*	2.1*	–	–
SR-XRF	Ink	11.90 (2)	0.20 (3)	2.81 (9)	1.18 (8)	0.48 (5)	0.41 (5)
SR-XRPD	Ink	10.7 (2)	ND	3.2 (3)	ND	–	–

*RDS < 5%.

### Ink powder characterization

**Figure 2:**
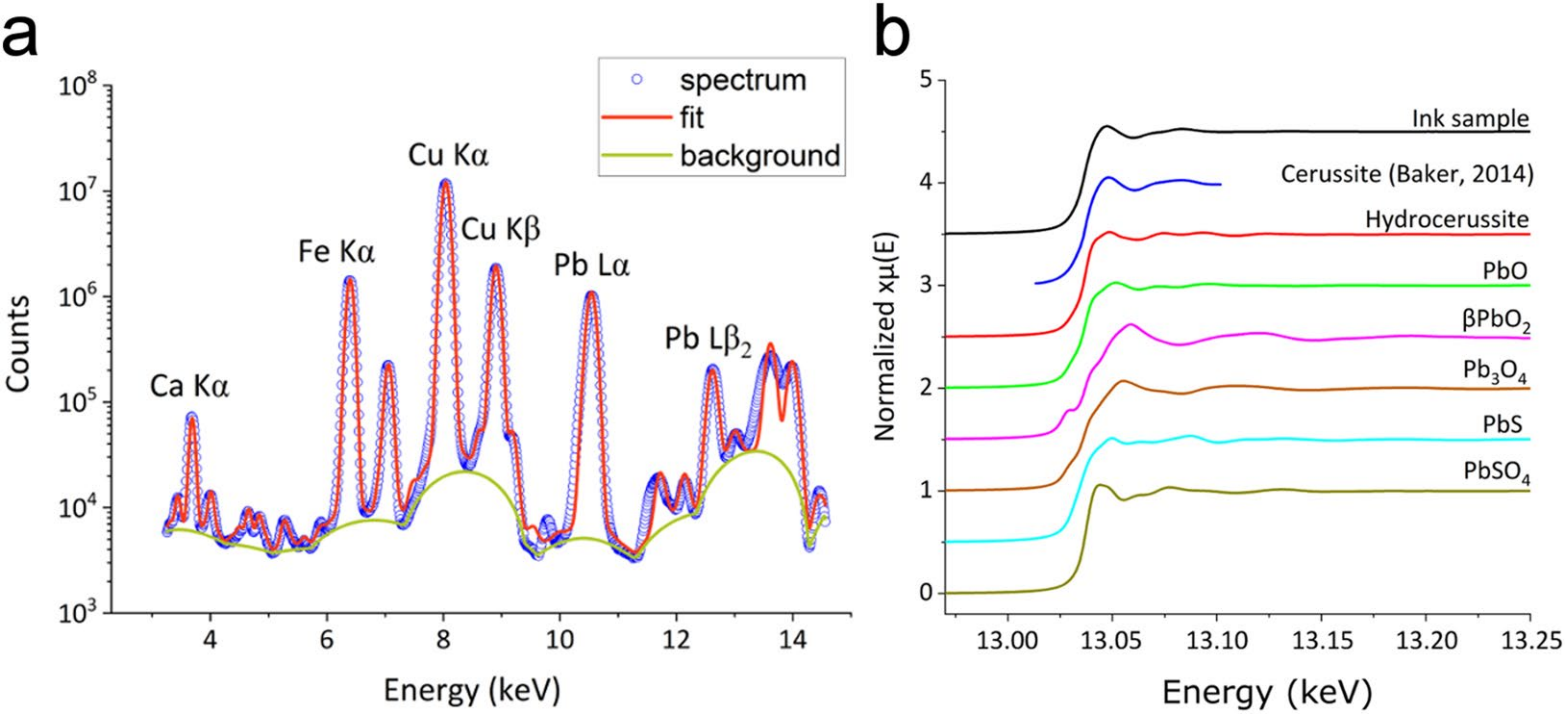
(**a**) SR-XRF average spectrum of the ink sample (experimental: empty blue circle) and its fit obtained with PyMca (red curve); (**b**) Normalized spectra of the ink sample and all the collected XAS data of reference compounds. The cerussite (PbCO_3_) spectrum is taken from Baker et al^22^.

The elemental composition of the black powder was obtained by means of SR-XRF. Since, on visual inspection, the ink powder showed great heterogeneity in the particle size, fluorescence spectra have been collected at different points of the sample. The spectra obtained did not show appreciable differences in terms of distribution of the elements or intensity of the peaks (i.e. element concentration). For this reason, the quantitative analysis has been performed on an average spectrum (Fig. 2a). An important fraction of the sample was composed of light elements like Si, Mg, Al and C which could not be quantified as they are not accessible with the set-up used for the experiment. Therefore, according to the phase and elemental quantification from other techniques (discussed below), the raw weight percentages were rescaled. The estimated values of the most representative elements of the ink powder are reported in Table 2. To confirm the results, Inductively Coupled Plasma-Optical Emission Spectrometry (ICP-OES) was also exploited. The most relevant elements quantified (15% of Cu, 3.5% of Pb and about 2% of Fe) are well in agreement with SR-XRF results (See Table 2). Several other elements in trace amounts, such as Mn, As, Zn, Ba and so on (See Table 3 for more details), have been revealed and quantified by Inductively Coupled Plasma Mass Spectrometry (ICP-MS). Some of them well matched those detected in the inkwell alloy by HHXRF. These evidences support the hypothesis of their presence in the ink powder as by-products of metallurgic works in the furnace.

**Table 3:** Trace elements (in ppm) revealed by ICP – MS in the ink powder.

	Mn	As	Zn	Ba	V	Sr	Ni	Cr	Co
ICP-MS Traces elements (ppm)	301	257	200	137	80.7	69.1	19.0	15.2	5.45

As mentioned above some authors reported that the co-presence of Cu, Fe and Pb could be compatible with a mixed ink, produced by intentionally mixing soot or charcoal with copper, iron and lead bearing minerals. The hypothesis is that metallic compounds, such as Pb, might have been added in order to enhance the black pigmentation. As reported in literature^6–8,18^, one of the more plausible Pb-based black pigments was galena (PbS).

Nevertheless, the present elemental analysis was not sufficient to attribute the ink powder to the mixed-ink family, and, therefore, a more accurate analysis on the source of Pb was necessary. With this aim, X-ray absorption spectroscopy (XAS) at the Pb L_3_-edge was performed. In Fig. 2b, the XANES spectra of the ink are reported, along with the spectra of other Pb-bearing minerals such as PbO (litharge), Pb_3_O_4_ (minium), Pb_3_(CO_3_)_2_(OH)_2_ (hydrocerussite) and PbCO_3_ (cerussite), that are known to be used as pigments or in cosmetics production^19,20^. In addition, also PbS (galena), β-PbO_2_ (Plattnerite) and PbSO_4_ (anglesite), well documented as pigment and degradation products of Pb-bearing pigments^18,21^, were used as references. In absence of a cerussite sample, its spectrum was taken from literature^22^. From Linear Combination Fitting (LCF) analysis (see Supplementary Fig. S2 online), using the spectra from the model compounds, it was possible to conclude that almost all the Pb in the sample is in the cerussite phase (84 ± 9% cerussite; 12 ± 8% anglesite; 4 ± 4% minium). Cerrusite and hydrocerrusite represent some of the most common products of metal corrosion in presence of carbonates^23^. The very high exchange rate of lead with carbonates, along with the high Pb concentration found in the inkwell (revealed by HHXRF) made us suspicious that the lead present in the ink powder, mainly in form of cerussite, could be ascribed to a degradation of the inkwell itself, and not to a mixed nature of the ink black powder. Even if the use of PbS to reinforce the black pigmentation of our ink could not be completely ruled out by our analyses at this stage, it is to be considered quite improbable. Indeed, as reported by Wagner et al., in an oxidative environment, galena turns into PbSO_4_^18^, a greyish-white pigment, that has been found by the XAS analysis. However, in such case we would expect to find anglesite in a higher amount in the ink, and clearly detectable also by other techniques. Only in a single case, Scanning Electron Microscopy coupled with Energy Dispersive X-ray spectroscopy (SEM–EDX) identified a fragment characterized by the co-localization of S and Pb, that could suggest the presence of PbSO_4_. Additionally, SEM–EDX measurements revealed a green flake to be mainly composed by Cu, attributable to malachite, co-localization of chromium and iron in an orange flake, probably chromite, a chromium-iron oxide (see Supplementary Fig. S3 online) and a thin metallic flake to be composed of Cu and Zn (see Supplementary Fig. S4 online). They could represent other corrosion products of the inkwell or impurities deriving from the metallurgy, glaze and glass productions of the furnaces^14,17^.

**Figure 3:**
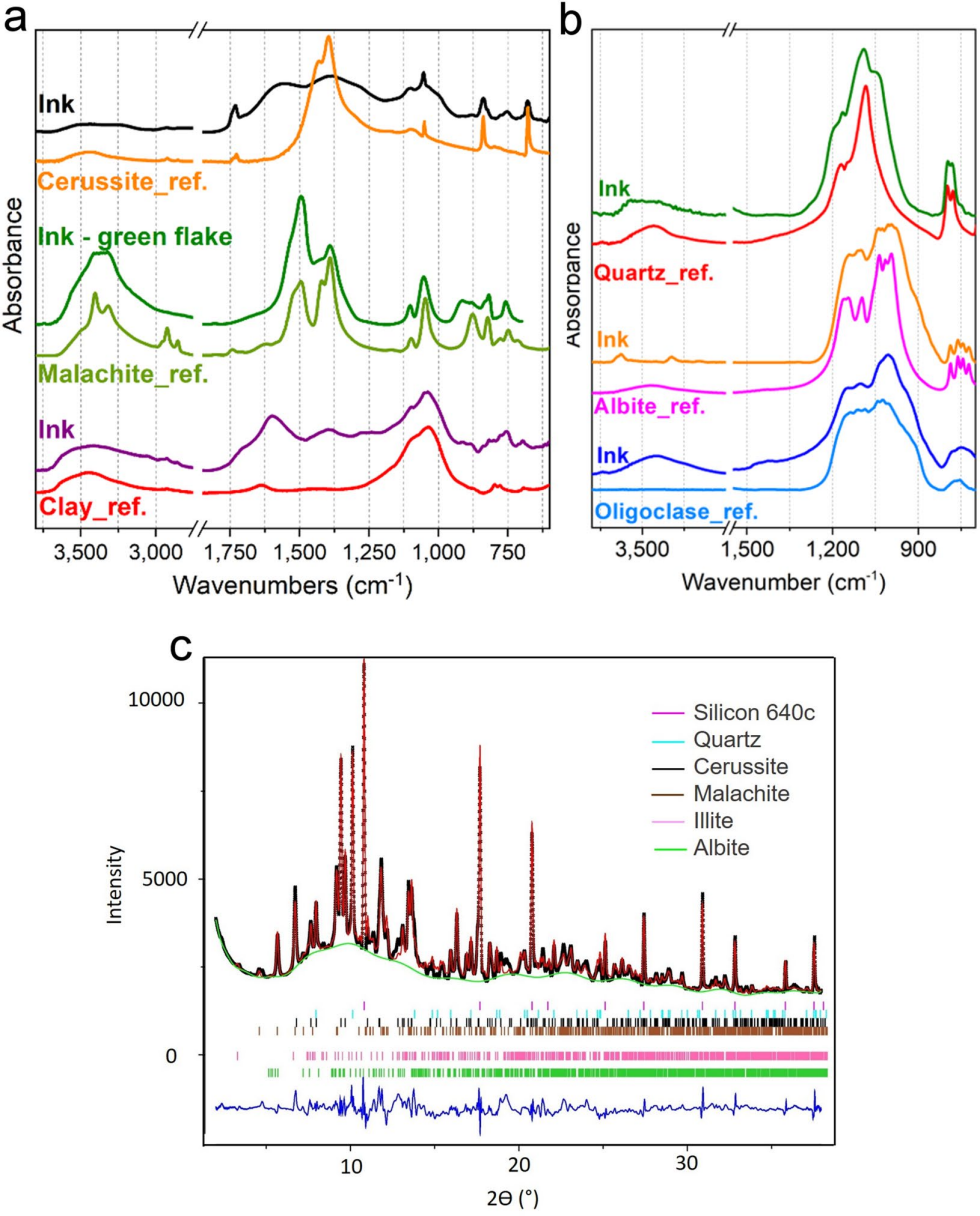
(**a,b**) Infrared spectra of several ink samples and possible reference spectra from the database (Kimmel Center for Archaeological Science Infrared Standards Library, https://www.weizmann.ac.il/kimmel-arch/infrared-spectra-library); (**c**) XRPD Rietveld refinement profile fit of the ink sample: black crosses are the experimental data, in red the calculated pattern. The residuals are displayed on the bottom in blue and the reflection ticks of each phase with the colours reported in the legend.

The presence in the black powder sample of cerussite (PbCO_3_) (black and orange infrared spectra in Fig. 3a) and malachite (Cu_2_(OH)_2_CO_3_) (green and light green infrared spectra in Fig. 3a) has been further confirmed by SR-Fourier-Transform Infrared (FTIR) microscopy, focusing the vibrational analysis on black and green flakes, respectively. Silicates are also very well represented in the sample, as shown by the comparison of an ink spectrum with a clay reference in Fig. 3a (purple and red line, respectively). In particular, some of the most common soil minerals, such as quartz, albite and oligoclase were identified in the grey flakes of the sample (Fig. 3b). These findings are well supported by the quantitative phase analysis of SR-X-Ray Powder Diffraction (XRPD) pattern (Fig. 3c). It showed the presence of cerussite (PDF 01–076-2056), malachite (PDF 01–076-0660), illite (PDF 00–002-0050), quartz (PDF 01–079-1906) and albite (PDF 01–076-0898) with percentages around 4.1(± 3), 18.7 (± 2) %, 29.9 (± 2) %, 9.8 (± 3) % and 3.9 (± 3) %, respectively. The Pb and Cu content in cerussite and malachite together with their weight fraction allowed to calculate the amounts of Pb (3.2%) and Cu (10.7%), which are in very good agreement with the results obtained by the ICP-OES and SR-XRF analysis (Table 2). We can, therefore, conclude that crystalline phases of the Cu and Pb carbonates are the principal source of Cu and Pb. Since both minerals represent the most common products in corrosion processes of lead and copper, their presence strongly supports the hypothesis that these metals found in the ink derive from the degradation process of the inkwell^16^ in the equilibrium exchange with carbonate components of the burial soil. Together with carbonates, the other crystalline phases identified represent the most common minerals in soils and, thus, they can be considered as a contamination deriving from the burial environment.

Nevertheless, the crystalline fraction of the sample is about 66,5%, whereas the amorphous part is around 33,4%, which is a not negligible fraction. Thermogravimetric Analysis (TGA) in static air (see Supplementary Fig. S5 online) shows a high inorganic residue, around 69%, and confirms a weight loss of about 31%, imputable to an organic part. This result strongly agrees with the amorphous content determined by the XRPD and suggests that the amorphous fraction is mainly of organic nature. However, a contribution of inorganic components in the amorphous phase cannot be excluded. In fact, both SR-XRF and ICP-OES revealed the presence of Fe (around 2%). Fe can therefore be present in the sample as oxides, as also corroborated by FTIR spectroscopy. Some FTIR spectra showed intense peaks at around 532, 469 and 429 cm^-1^ that could be attributed to iron oxides^24^ (see Supplementary Fig. S6 online).

Infrared and UV Resonant Raman spectroscopy are crucial techniques for detecting the presence and unveiling the nature of amorphous-organic compounds where other techniques are completely blind.

**Figure 4:**
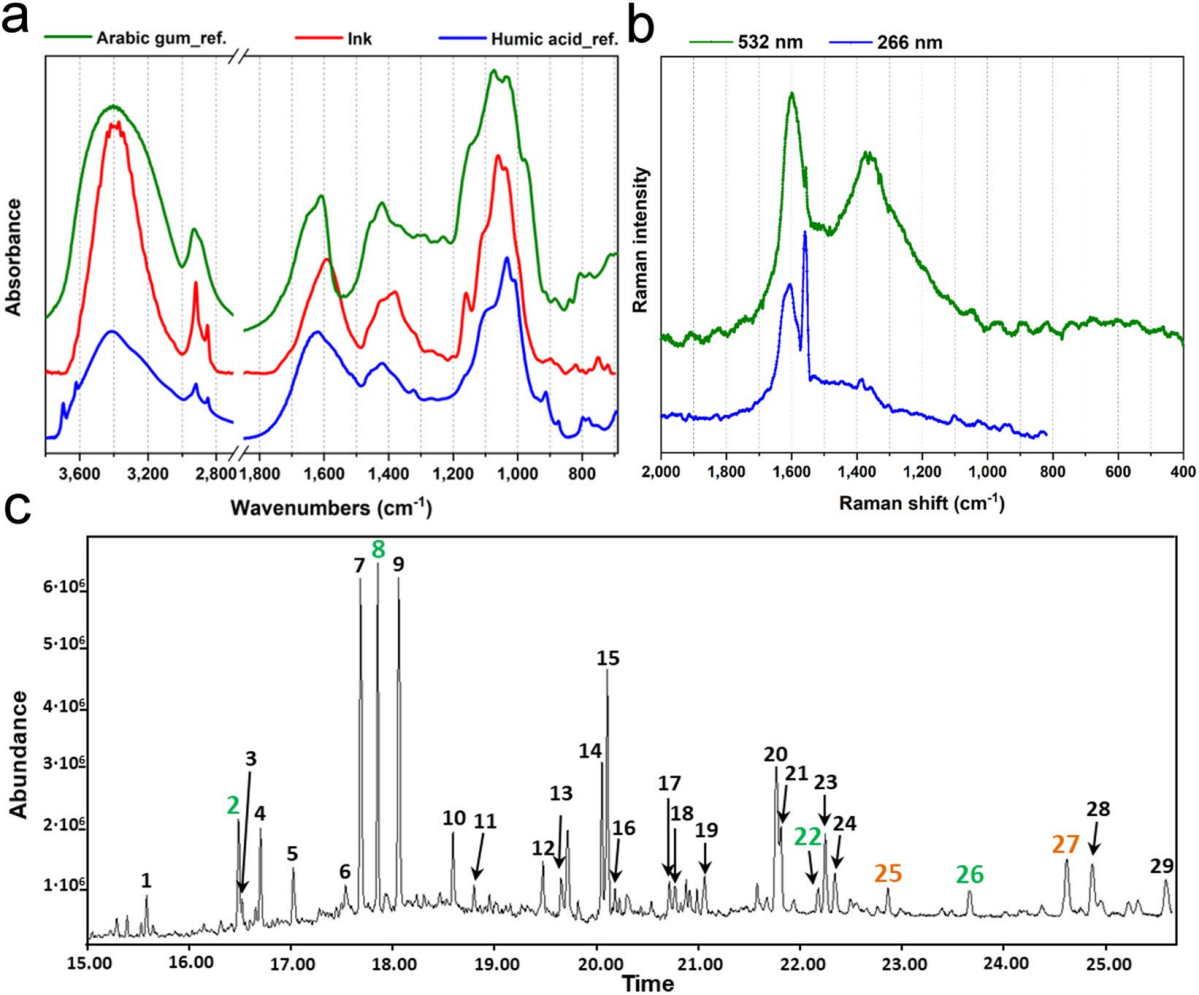
(**a**) Infrared spectrum of an ink sample and possible reference spectra from the database (Kimmel Center for Archaeological Science Infrared Standards Library, https://www.weizmann.ac.il/kimmel-arch/infrared-spectra-library); (**b**) Raman spectra collected by 532 nm and 266 nm laser, respectively; (**c**) chromatogram: the main peaks are indicated by numbers and reported in Table 4. Black numbers indicate combustion products, green numbers indicate vegetable origin compounds, orange numbers indicate compounds of both vegetable and/or animal origin.

Figure 4a shows an additional IR spectrum of the ink (red line) where quite intense and sharp peaks at around 2922 and 2853 cm^-1^ were detected. They are due to CH_2_ asymmetric and symmetric stretching, respectively, and are indicative of the presence of an organic fraction. At lower wavenumbers two broad and intense bands at around 1600 and 1400 cm^-1^ can be observed. They can be attributed to the asymmetric and symmetric stretching of the COO^-^ groups and could be associated with carboxylate fractions. The positions of carboxylate peaks strongly depend on the coordinated cations and, therefore, the band broadening reflect the complex mineral composition of the burial soil^24^. The band at 1600 cm^-1^ is also accompanied by a shoulder around 1710 cm^-1^. It is due to the C=O stretching mode of esters, ketones or aldehydes. The infrared features just described could be indicative of the presence of a degraded organic substance. For the purpose of comparison, the reference spectrum of a humic acid (with traces of kaolinite), a compound deriving from organic matter degradation^25^, is shown in Fig. 4a (blue line). Moreover, the band at 1600 cm^-1^ could also be due to the C=C stretching vibration of aromatic rings and it could represent the chemical fingerprint of combustion products deriving from wood burning performed in antiquity to obtain black carbon. Finally, the very intense and broad band in the range 1200–900 cm^-1^, even if partially influenced by the signal of silicates, could also suggest the presence of carbohydrates, the class of compounds to which Arabic gum belongs^24^ (See for comparison the green spectrum of Arabic gum in Fig. 4a).

Figure 4b reports the Raman spectra collected on the sample, employing excitation wavelengths of 532 nm and 266 nm. The latter was added as the measurement at 532 nm alone is not sufficient to confirm the presence of amorphous carbon, while the UV excitacition is more sensitive to carbon components with respect to Raman in visible range. The observation of the typical G and D1 bands, centered respectively at 1600 cm^-1^ and 1360 cm^-1^ confirms the presence of amorphous carbon. In particular, the position of the D1 band, which is the same at 532 nm and 266 nm, suggests that such amorphous carbon is mainly composed by a low-sp3 hybridization structure or nano-crystalline graphite^26^. Composites with high sp3 hybridization can be excluded. Nevertheless, comparison of the G and D1 peak positions with the scientific literature ^27^ suggests the presence of a so-called black earth (a mix of black chalk, low-ordered graphite, quartz, iron oxides and other minerals). We believe that this result should be more safely associated to the contamination by the burial soil.

For a more detailed and conclusive analysis of the amorphous-organic phase of the ink powder, Gas-Chromatography coupled with Mass Spectrometry (GC–MS) analysis was performed. It revealed that the ink powder is characterized by 29 organic compounds (See Fig. 4c and Table 4): 2 fatty acids, 22 polycyclic aromatic hydrocarbons (PAHs), 4 long chain n-alkanes and 1 heterocyclic aromatic compound. The large amount of PAH and the presence of the heterocyclic aromatic could derive from a biomass combustion process^28^ (peaks labelled by black numbers in Fig. 4c). Long chain n-alkanes with an even number of carbon atoms (n-triacontane and n-dotriacontane) can be of both animal and vegetable origin (peaks labelled by orange numbers in Fig. 4c) and therefore their attribution is uncertain: they can represent part of the original organic ink matrix or contaminations from the burial environment. The presence of traces of fatty acids (hexadecanoic and octadecanoic acid) and long chain n-alkanes with an odd number of carbon atoms (n-nonacosane and n-hentriacontane) suggest the presence of a vegetable matrix (peaks labelled by green numbers in Fig. 4c). In particular, n-hentriacontane is a constituent of Arabic gum (PubChem CID: 12,410). This finding strengthens and ultimately confirms all the results obtained by Resonant Raman and FTIR spectroscopy. As mentioned above, Table 1 summarizes the main results of our research, highlighting the specific contribution of each technique.

**Table 4:** List of organic compounds identified by GC–MS analysis (Peak number refers to labels in Fig. 4c).

Peak n°	Retention time (min.)	Compound	CAS	Identification	Class	Attribution
1	15,5761	Phenanthrene	85-01-8	MS, RT	PAH	Biomass combustion
2	16,4813	Hexadecanoic acid, methyl ester	112-39-0	MS, RT	Fatty acid (as methyl ester derivative)	Vegetable matrix
3	16,5102	Anthracene, 2-methyl-	613-12-7	MS, Lee	PAH	Biomass combustion
4	16,6932	Phenenthrene, 1-methyl	832-69-9	MS, Lee	PAH	
5	17,0159	Naphthalene, 2-phenyl-	612-94-2	MS, Lee	PAH	
6	17,5311	Quinoline, 6-phenyl-	612-95-3	MS, Lee	Heterocyclic aromatic (origin: coal tar)	
7	17,6804	Fluoranthene	206-44-0	MS, RT	PAH	Biomass combustion
8	17,8489	Octadecanoic acid, methyl ester	112-61-8	MS, RT	Fatty acid (as methyl ester derivative)	Vegetable matrix
9	18,0511	Pyrene	129-00-0	MS, RT	PAH	Biomass combustion
10	18,5856	Phenanthrene, 1-methyl-7-(1-methylethyl)-	483-65-8	MS, Lee	PAH	
11	18,7975	Pyrene, 1-methyl-	2381-21-7	MS, Lee	PAH	
12	19,4668	11H-Benzo[a]fluoren-11-one	479-79-8	MS, Lee	PAH	
13	19,6498	7H-Benzo(c)fluoren-7-one	6051-98-5	MS, Lee	PAH	
14	20,0495	Benzo(a)anthracene	56-55-3	MS, RT	PAH	
15	20,1025	Chrysene	218-01-9	MS, RT	PAH	
16	20,1795	Benzo[a]pyrene, 4,5-dihydro-	057,652-66-1	MS, Lee	PAH	
17	20,714	Benz[a]anthracene, 1-methyl-	2498-77-3	MS, Lee	PAH	
18	20,767	Benz(A)anthracene-7,12-dione	2498-66-0	MS, Lee	PAH	
19	21,0559	2,2′-Binaphthalene	612-78-2	MS, Lee	PAH	
20	21,7637	Benzo(b)fluoranthene	205-99-2	MS, RT	PAH	
21	21,8071	Benzo(k)fluoranthene	207-08-9	MS, RT	PAH	
22	22,173	n-Nonacosane	630-03-5	MS, RT	Long chain n-alkane	Vegetable matrix
23	22,2453	Benzo[e]pyrene	192-97-2	MS, RT	PAH	Biomass combustion
24	22,3416	Benzo(a)pyrene	50-32-8	MS, RT	PAH	
25	22,8279	n-Triacontane	638-68-6	MS, RT	Long chain n-alkane	Animal/ vegetable origin—contamination
26	23,6658	n-Hentriacontane	630-04-6	MS, RT	long chain n-alkane	Vegetable matrix/ Arabic Gum
27	24,6192	n-Dotriacontane	544-85-4	MS, RT	long chain n-alkane	Animal/ vegetable origin—contamination
28	24,8696	Indeno(1,2,3-cd) pyrene	193-39-5	MS, RT	PAH	Biomass combustion
29	25,5919	Benzo[ghi]perylene	191-24-2	MS, RT	PAH	

## Conclusions

The black ink powder found inside a bronze inkwell of the I century AD was characterized by a multi-analytical approach with a sequence of non-destructive and SR-based techniques as well as by conventional destructive methods.

In the first instance, the co-presence of Pb, Cu and Fe, revealed by SR-XRF and ICP-OES, induced us to hypothesize that the black powder could belong to the mixed-ink family, for which metals were added on purpose in the formulation. Nevertheless galena (PbS), the most probable Pb-based black pigment reported in ink formulation, was not detected by XANES analysis, while PbSO_4_, the most probable oxidative degradation product of PbS, was revealed only as minority constituent. Instead, cerussite and malachite, carbonates of Pb and Cu respectively, are indeed the most abundant crystalline constituents of the ink, as recognized by XANES, SR-XRPD and SR-FTIR. They can be considered as degradation products of the bronze alloy of the inkwell, the composition of which was assessed by HHXRF. Actually, the analysis performed directly on the inkwell and its lid revealed that their bronze alloy is characterized by Cu, Sn, Pb and Fe. The reaction of Pb and Cu metals with the carbon dioxide, present in the air or contained in the circulating waters, or with carbonates, present in the soil of the burial environment, perfectly justifies the results. Indeed, a heavy contamination of the ink powder from the burial environment is also deducible by the presence of common minerals of the soil, such as quartz, albite, and illite, as recognized by SR-FTIR and SR-XRPD. Finally, iron, as oxides, and other in traces metals, quantified by ICP-MS, can be considered as ink impurities deriving from the metallurgic activities performed in the same furnace and/or corrosion products.

Nevertheless, the crystalline inorganic fraction does not represent the entire sample, while a large amorphous organic fraction must be considered as well. The combined use of FTIR, Raman, and GC–MS evidenced that the black powder is mainly composed by amorphous carbon deriving from the combustion of wood and traces of substances of vegetable origin. Among them, a marker of Arabic gum was found. This allowed us to unequivocally identify the dry black powder as a writing ink.

Summarizing, the present work emphasizes the importance of using multi-instrumental and highly sensitive methods, like SR-based techniques, to investigate complex samples, such as precious Cultural Heritage goods. In particular, the fruitful collaboration among many experts gave us the opportunity to perform measurements not only on the ink powder, but also on its ancient container that allowed us to observe all the degradation products of the bronze alloy and to postulate their possible migration and ultimate contamination of the ink powder. This approach was fundamental in order to avoid a misleading interpretation of the results that could occur as a consequence of a non-exhaustive characterization of the sample.

## Methods

### Characterization

Before the restoration, 40 mg of black powder was scraped with the tip of a scalpel and stored in a clean/sterile vessel. The powder shows a deep black colour with some micrometric green, grey and orange flakes and a heterogeneous particle size (Fig. 1d). The characterization of the ink powder was done by using a multi-technical approach.

### SR-FTIR analyses

were performed at the Chemical and Life Sciences branch of the SISSI beamline^29^. A small amount of sample has been grinded and divided into 4 portions. Each portion has been measured separately in the sample compartment of the Vertex 70v interferometer, in the closed diamond compression cell (Diamond EX press by S.T. Japan, clear aperture 2 mm), using a 5X focusing unit (A524/Q, Bruker Optics) and the Bruker wide range components (i.e. beamsplitter and DTGS detector) for covering FIR (Far-Infrared) and MIR (Mid-Infrared) spectral regions in a single scan. Each spectrum was collected averaging 128 scans at 4 cm^-1^ spectral resolution. Indeed, extending the spectral range from 4000 to 120 cm^-1^ allows better highlighting the presence of metal–organic spectral features. The collected spectra were baselined and averaged. After the measurements in the sample compartment, the diamond compression cell has been opened and the samples were measured in transmission mode on half compression cell by a Vis-IR Bruker Hyperion 3000 microscope coupled with the same interferometer in the MidIR range (MCT-A detector, 4000–700 cm^-1^). Around 10 spectra for each sample were acquired averaging 128 scans at 4 cm^-1^ spectral resolution, setting lateral resolution at 30 × 30 μm carefully selecting the sample areas according to the observable differences in colour. In addition, also some green, grey and orange flakes have been selected from the black ink and analyzed after flattening on the opened diamond compression cell using the same experimental conditions as described above.

For the assignments, all of the acquired FTIR spectra were compared with that reported in the literature and IR spectral libraries (Kimmel Center for Archaeological Science Infrared Standards Library, https://www.weizmann.ac.il/kimmel-arch/infrared-spectra-library and IRUG Spectral Database, www.irug.org).

Some ink samples were peeled off with carbon conductive adhesive tape from the culet of the diamond after FTIR spectromicroscopy analysis and S**EM/EDX measurements** were performed using a Zeiss Supra 40 field emission gun, an SEM equipped with a Gemini column and an in-lens secondary electron detector operated at 5 kV. EDX analyses were performed using a LN2-free X-Act Silicon Drift Detector (Oxford X-ray detection system, Aztec EDS). SEM/EDX measurements were performed at the IOM-CNR laboratories. The procedure used has been described by Sano et al.^30^.

In order to detect and quantify major- and trace elements, an ink sample (14 mg) has been solubilized by microwave assisted (Multiwave PRO, Anton Paar) acid mineralization. Then, the acid solution has been diluted to 10 mL with milliQ water and filtered with 0.45 µm syringe filter (GHP, Acrodisc, Pall).

Major elements (Ca, Mg, Si, Pb, S, P, Cu, Pb, Fe, Al) in the acid solution were detected by means of Inductively Coupled Plasma Optical Emission Spectrometry (**ICP-OES**), while trace elements (As, Ba, Co, Cr, Cs, Mn, Ni, Sr, V, Zn) were detected by means of Inductively Coupled Plasma Mass Spectrometry (**ICP-MS**). Instruments and methods used were described by Pavoni et al. ^31^.

The precision of the measurements as relative standard deviation (RSD %) for the analysis was always less than 5% for all the elements.

**TGA-DTA** has been performed on 30 mg of the black powder; with a: Netzsch STA 409. Analyses were done in static air and the temperature was increased from 24 °C to 600° C with a heating rate of 10 °C/min.

**GC–MS** 9 mg of the sample was extracted in 1 ml of 1:1 cyclohexane/heptane mixture using an ultrasonic bath for 1 h. Then 0.5 ml of 2 M KOH in methanol was added to convert fatty acids to their respective methyl esters. The mixture was centrifuged and the upper phase filtered on 0.45 μm PTFE membrane filters. The filtered phase was concentrated to 200 μl by a nitrogen stream and 1 μl was injected into the GC–MS system (Agilent 6890/5973 Inert, Agilent DB 5 ms UI capillary column: 30 m × 0.25 mm i.d. × 0.25 µm film thickness) with helium as carrier gas. The system was equipped with an autosampler (Gerstel MPS2). The injection port was set at 300 °C, the GC oven temperature program was: 55 °C to 150 °C at 10 °C/min; hold for 1 min; 150 °C to 300 °C at 15 °C/min; hold for 5 min. The MS m/z range was set from 35 to 450 u.

The compounds were identified by a software match with the NIST mass spectra database and using FAME standard (FAME Mix C8-C24 CRM by Supelco), n-alkane standard (Alkane standard solution C_21_-C_40_ by Sigma-Aldrich) and PAH standard (Custom PAH Solution, 22–1 by o2si Smart Solutions) or, for other PAHs, by match with Lee's retention index^32^ NIST database (https://webbook.nist.gov/chemistry/).

### Raman measurements

have been carried out at the IUVS beamline. A complete description of the experimental apparatus can be found in D’Amico et al.^33^.

266 nm and 532 nm conventional laser sources have been used as excitation sources. The beam reaching the sample was approximately 300 μW. We apply a continuously oscillating horizontal movement on the sample surface in order not to expose always the same sample portion to the laser beam. This has been demonstrated to be sufficient, in coupling with a sample movement procedure, to avoid photo-degradation^26^. The Raman scattering signal was collected with a backscattering configuration. A Czery-Turner spectrometer with a focal length of 750 mm, coupled with a holographic reflection grating of 1800 g/mm and with a Peltier-cooled back-thinned CCD was employed to get the Raman signal. Spectral resolution was set to 8 cm^-1^. Raman frequencies were calibrated by means of cyclohexane spectra^34^.

### SR-XRF measurements

were performed at X-Ray Fluorescence beamline^35,36^ in 45/45° geometry at 14 keV, and therefore above the Pb-L_3_ edge, in high vacuum conditions (10^–8^ mbar), using a high energy multilayer. The beam spot size was of 50 × 50 µm^2^ and the XRF data were collected using a Bruker XFlash 5030 Silicon Drift detector (SDD) mounted under 90° with respect to the primary X-ray beam. XRF spectra were processed using the open source software PyMca^37^. The elemental quantification was obtained through a fundamental parameters fitting strategy.

### Hand-Held XRF

Spectrometer Niton XL3t Goldd by Thermo-Scientific was used for the qualitative elemental analysis of the inkpot preserved in the museum. The spectrometer consists of a low power (2 W) x-ray tube with a Ag anode and 50 kV maximum voltage and a silicon drift detector. The instrument can provide elemental concentrations using built-in calibrations which are using different combinations of tube voltage and filters on primary radiation to optimize the sensitivity for different ranges of X-ray energies; for these measurements, the “general metal” calibration was used. For small size samples like the inkwell, measurements can be better performed using a dedicated support, which provides more “stable” and reproducible conditions.

### SR-XRPD measurements

have been performed at the Elettra x-ray diffraction beamline (XRD1)^38^. Data were collected in transmission mode packing the powder in a borosilicate capillary with a diameter of 300 μm. A pattern was collected at room temperature using a monochromatic wavelength of 0.700 Å (17.71 keV), 200*200 μm^2^ spot size, on a hybrid-pixel Dectris Pilatus 2 M area detector (Dectris Ltd., Baden-Daettwil, Switzerland). The pattern was then integrated using the Fit2D program ^39^ after calibrating the hardware setup with LaB_6_ standard reference powder (NIST 660a). Before the XRPD measurement 10wt% of a standard powder (Silicon 640c-Nist) was mixed to the ink powder to calculate the amorphous amount eventually present in the sample.

The quantitative phase analysis (QPA) was performed by means of the Rietveld method using the GSAS package^39^.

Pb L_3_-edge **XAS** spectra were collected at the XAFS beamline^40^ in transmission mode using a fixed exit double crystal Si (111) monochromator. For all the measurements, energy calibration was accomplished by collecting simultaneously a reference spectrum of metallic Pb placed in a second experimental chamber after the sample and after the I1 ionization chamber, with the position of the first inflection point taken at 13,035.0 eV.

All spectra were collected at room temperature and in vacuum conditions, with a variable step as a function of the energy: Large steps (5 eV) in the first 200 eV of the spectrum, smaller steps (0.2 eV) in the XANES region and k-constant steps of 0.03 Å-1 (up to 1.8 eV) in the EXAFS region.

For the ink sample, 7 spectra have been collected and merged in order to increase the signal to noise ratio, whereas for the reference compounds 3 spectra were enough. Background removal, normalization of XANES spectra as well as LCF were performed using the Athena software package^40^.

## Supplementary Information

Supplementary Information

## Data Availability

The datasets generated and analyzed during the present study are available from the corresponding authors on reasonable request.
