# Supplementary material for: A multidisciplinary study unveils the nature of a Roman ink of the I century AD
Source: Sci Rep. 2021 Mar 31;11:7231. doi: 10.1038/s41598-021-86288-x (PMC8012642; doi:10.1038/s41598-021-86288-x)
Supplement: Supplementary file 1 — Supplementary Information [file 41598_2021_86288_MOESM1_ESM.pdf]

SUPPLEMENTARY INFORMATION

A multidisciplinary study unveils the nature of a Roman ink of the I century AD

Mirta Sibilia[1], Chiaramaria Stani[2*], Lara Gigli[3], Simone Pollastri[3], Alessandro Migliori[1] Francesco D'Amico[3], Chiara Schmid[4], Sabina Licen[5], Matteo Crosera[5], Gianpiero Adami[5], Pierluigi Barbieri[5], Jasper R. Plaisier[3], Giuliana Aquilanti[3], Lisa Vaccari[3], Stefano Buson[6], Federica Gonzato[6*]

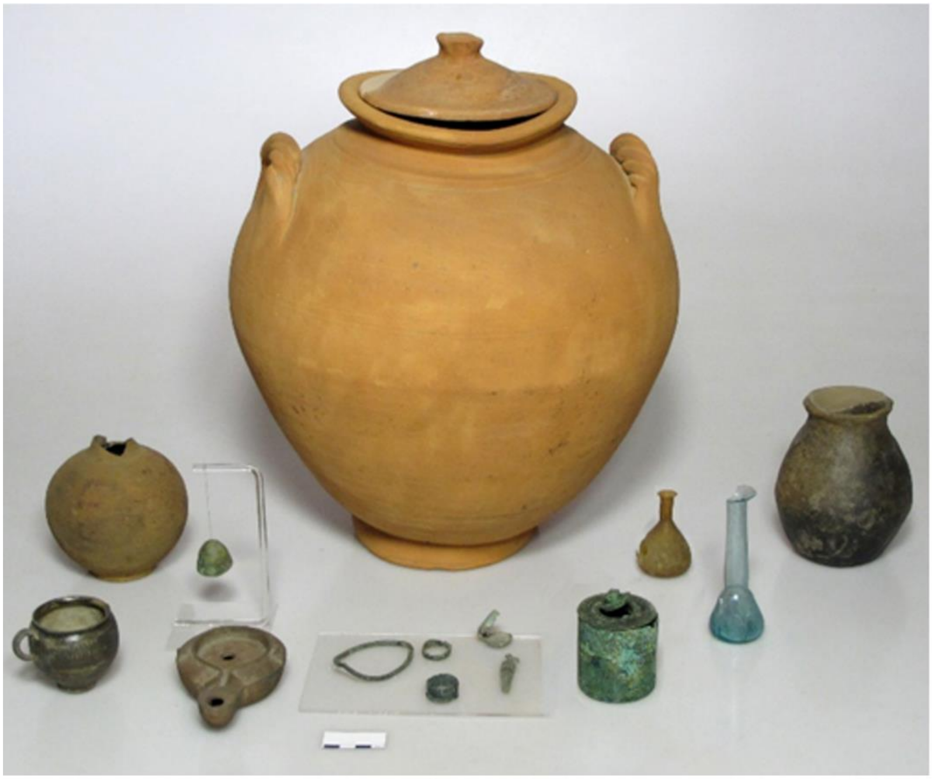

Supplementary Figure S1: Archaeological context. Grave goods of civic tomb XII, as exposed at the Museo Nazionale Atestino of Este, Padua, Italy. The grave good is composed by a big funerary clay urn, some jewels, a couple of *unguentaria,* some potteries and a lamp.

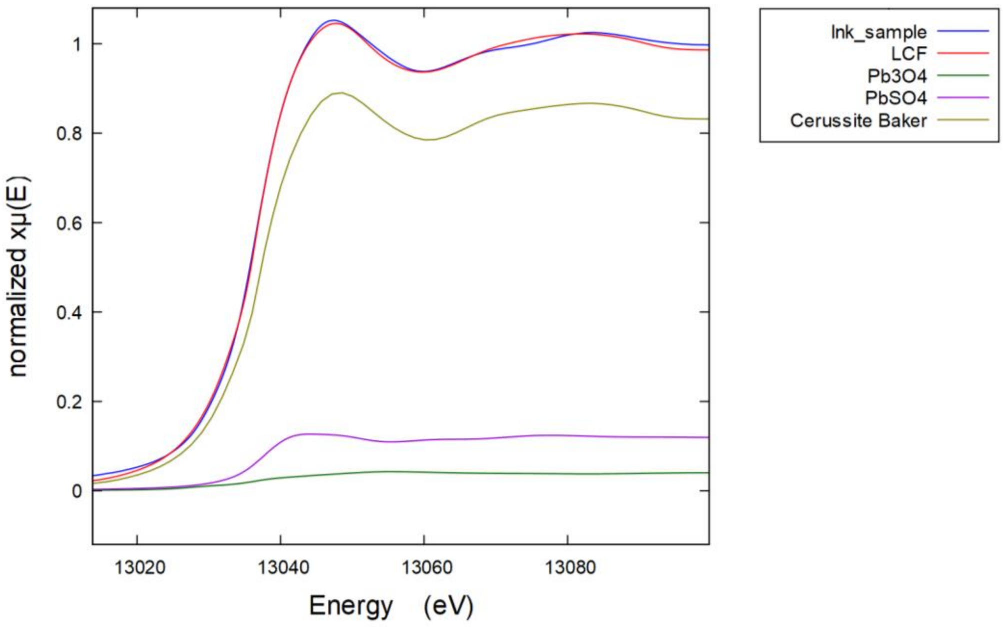

Supplementary Figure S2: XANES Linear Combination Fitting (LCF) analysis of the Ink spectrum performed using the Athena software package.

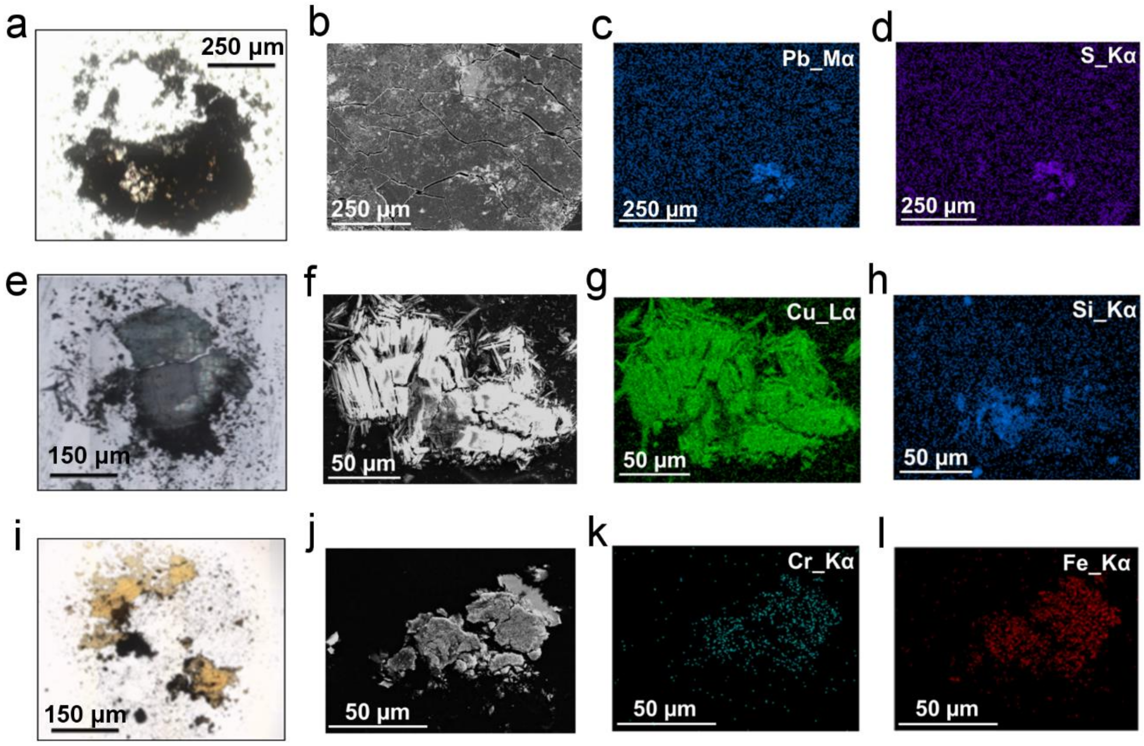

Supplementary Figure S3: a-b. optical and SEM images of an ink particle; c-d. EDX maps for Pb Mα and S Kα. Pb and S co-localize and can represent a trace of Anglesite ($PbSO_4$); e-f. optical and SEM images of a green flake; g-h. EDX maps for Cu Lα and Si Kα. The sample is mainly composed by Cu; i-j. optical and SEM images of an orange flake; k-l. EDX maps for Cr Kα and Fe Mα. Cr and Fe co-localize and can represent an iron chromium oxide (FeO,Cr_2O_3 or $FeCr_2O_4$).

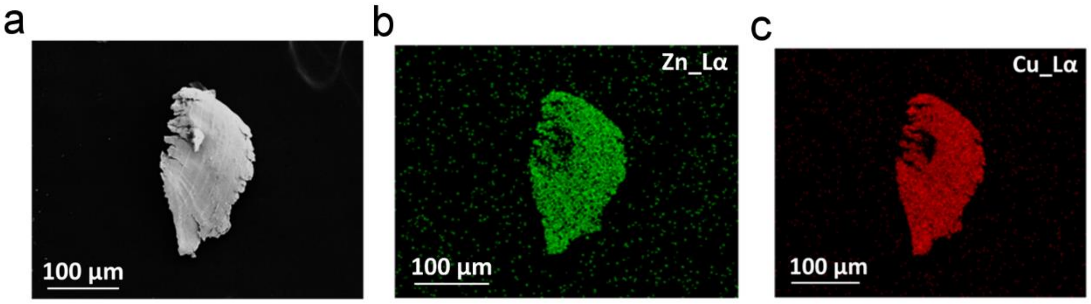

Supplementary Figure S4: a) SEM image of a metallic flake recovered in the ink powder; b-c) EDX maps for Zn Lα and Cu Lα.

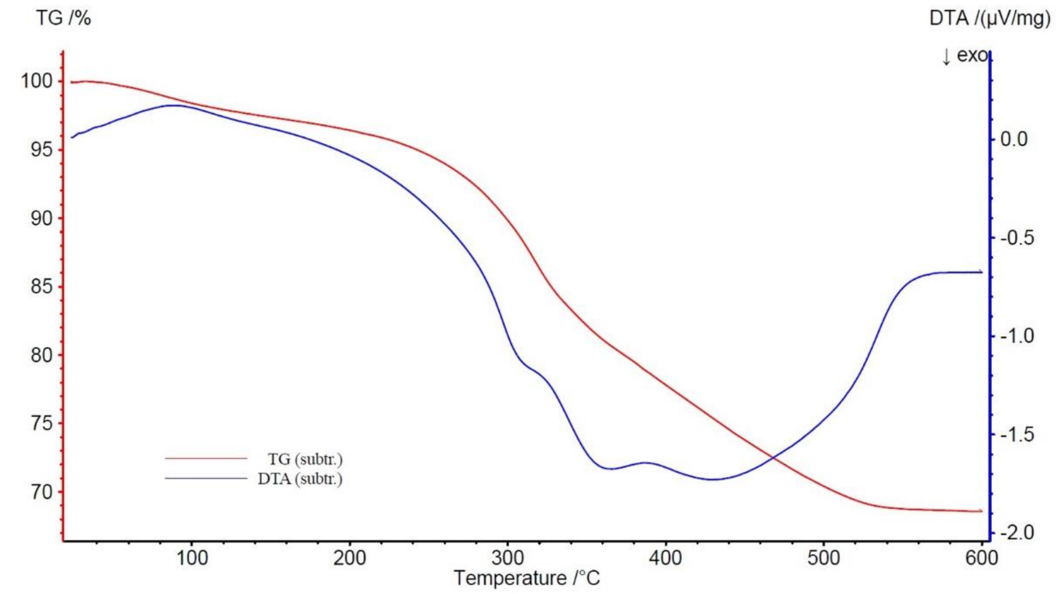

Supplementary Figure S5: TGA-DTA curves.

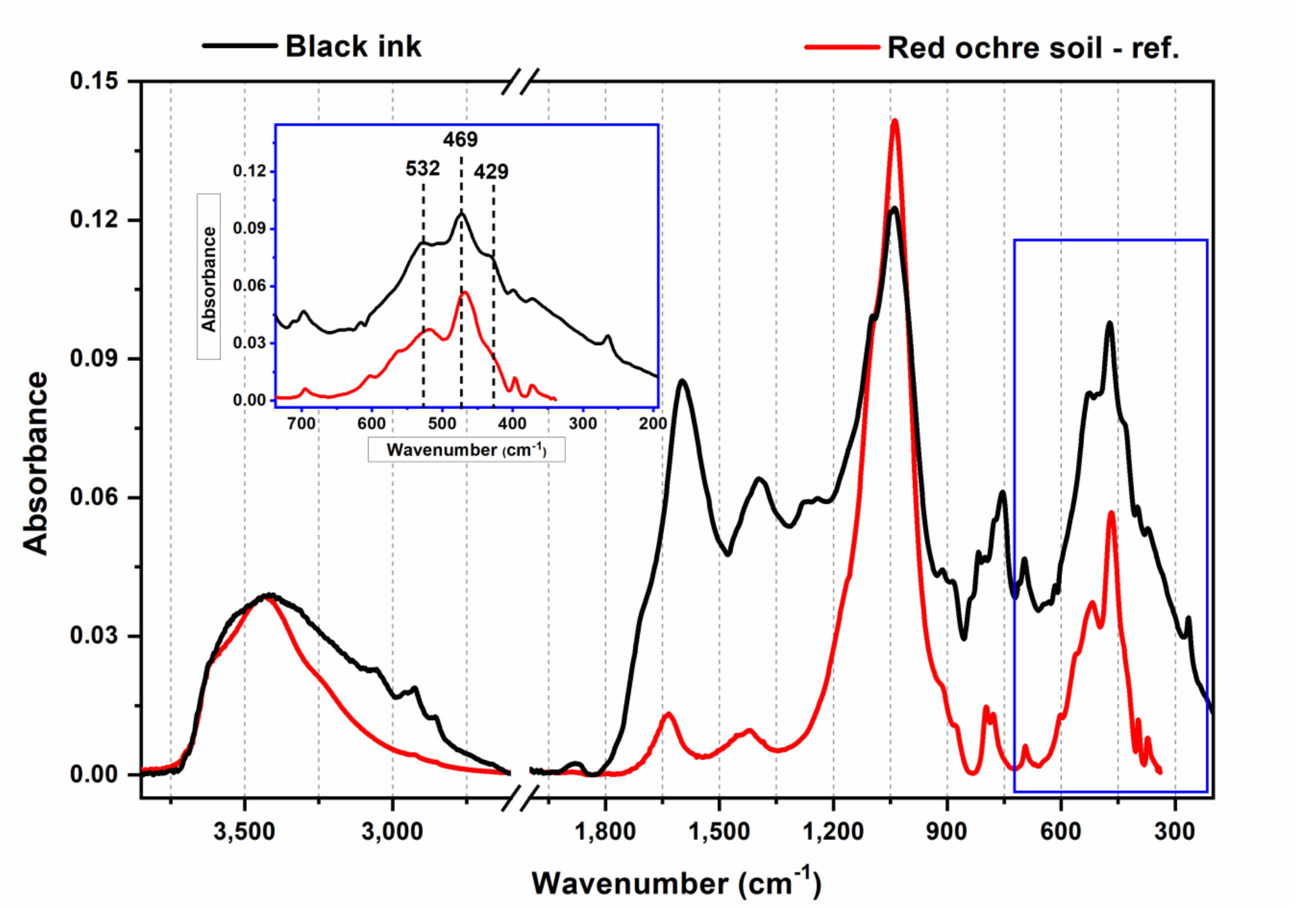

Supplementary Figure S6: Infrared spectra of ink (black line) compared with a red ochre reference spectrum. In the inset, the region at lower wavenumbers. The peaks attributed to the presence of iron oxides are highlighted.
